# Has local government debt crowded out enterprise innovation?

**DOI:** 10.1371/journal.pone.0277461

**Published:** 2022-11-22

**Authors:** Xiaoxu Zhang, Rongxue Jin

**Affiliations:** School of Public Finance and Taxation, Zhongnan University of Economics and Law, Wuhan, Hubei, China; Massey University - Albany Campus: Massey University - Auckland Campus, NEW ZEALAND

## Abstract

This paper tests the impact of local government debt on enterprise innovation based on 2011–2017 A-share non-financial enterprise data from Shanghai and Shenzhen Stock Exchanges. The results show that the relationship between government debt and enterprise innovation relationship follows an inverted U-shaped pattern. Endogeneity processing and robustness test result confirm the results of the model built for this study. Heheterogeneity analysis finds that the inflection points of local government debt in large enterprises, non-SOEs (non-state-owned enterprises) and poorly financialized regions are lower. Financing constraints and corporate profits play a part of the intermediary effect in the inverted U-shaped relationship between local government debt and enterprise innovation. Further research shows that Digital finance plays a moderating role in the impact of local government debt on enterprise innovation. Therefore, to keep local government debt scale compliant and to maximize the efficiency of digital finance are of great significance in terms of boosting enterprise innovation and improve economic development.

## 1. Introduction

With the transformation of China’s economic growth mode from focusing on speed to focusing on quality, innovation has become an important force to improve the quality of economic development in the context of the new normal for economic development. As the core and main body of innovation, Whether or not to stimulate the innovation vitality of enterprises is crucial to the success or failure of China’s innovation-driven development strategy. In recent years, with the support of a series of innovation reforms and innovation policies, enterprises have made remarkable achievements in innovation, but they still lag behind economic growth seriously and cannot meet the needs of high-quality economic development. The innovation activities of enterprises are carried out in a specific environment. As a premise for the survival and development of enterprises, the environment directly affects the decision-making behavior of enterprises, and further affects the innovation activities of enterprises [1]. As an important force affecting the environment, LGD inevitably has an important impact on the innovation activities of enterprises.

Some scholars have studied the relationship between local government debt and enterprise innovation. Xu et al. [2] analyzed the impact of local government debt on enterprise innovation by calculating the sum of short-term liabilities of LGFV, and found that there is a negative correlation between local government debt and enterprise innovation. Chen et al. [3] analyzed the impact of local government debt on enterprise green innovation by calculating the debt data of 30 provinces, and found that local government debt inhibited enterprise green innovation. Fan et al. [4] took the sum of bonds issued by LGFV and bank loans as the data of LGD, examined the impact of local government debt on enterprise innovation, and concluded that local government debt was not favorable to enterprise innovation. Croce et al. [5] and Ferraro and Peretto [6] also believed that local government debt would inhibit enterprise innovation. Based on different debt data, existing studies on the relationship between local government debt and enterprise innovation, have drawn the conclusion that local government debt is unfavorable to enterprise innovation. The main source of LGD is bank deposits, China’s financial system has always been dominated by large banks [7]. Excessive debt scale crowds out credit resources of enterprises, thus inhibiting enterprise innovation. However, economic growth, improvement of infrastructure and moderately rising of housing prices brought about by moderate local government debt have created a favorable condition for enterprises to invest in innovation. Therefore, there may be a nonlinear relationship between LGD and enterprise innovation. The existing research lacks a relatively complete analytical framework and empirical conclusions consistent with reality. Based on the theories and methods provided by existing achievements, this paper re-analyzes the relationship between LGD and enterprise innovation. The marginal contribution of this paper is mainly reflected in two aspects: First, this paper empirically verifies the nonlinear relationship between local government debt and corporate innovation, and analyzes the mediating effects of financing constraints and corporate profits. Second, considering the rapid development of digital finance and its importance in helping to solve the disadvantages of traditional finance, this paper further integrates digital finance into the relationship between LGD and enterprise innovation, and analyzes its moderating effect.

## 2. Theoretical analysis and the hypothesis of this research

This chapter seeks to assess how LGD will impact enterprise innovation from aspects of economic effect, local government debt investment and the increase of housing price.

First, the economic effect of local government debt. Moderate local government debt can stimulate investment and consumption, and promote economic growth, while higher local government debt will constrain private investment and induce financial risks catalyzed by inaapropriate interest rates or other means, hindering economic growth. Accordingly, the relationship between local government debt and economic growth follows an inverted U-shaped pattern [8, 9]. When economy grows vigorously, the enterprise records better profitable performance so the manager would be optimistic towards economic outlook and incline to invest into innovation. In contrary, enterprise does not perform well when economic growth is sluggish, so managers are overwelmed by pessimistic sentiment which inhibits the innovation investment [10]. Due to its close correlation with economic development, local government debt thus impacts enterprise innovation, which the relationship pattern is an inverted U-shaped curve.

The proceeds of local government debt are invested in infrastructure and industrial parks which can bring about economic agglomeration. Moderate economic agglomeration will improve the enterprise profitability. In the industrial cluster, enterprises enjoy the knowledge spillover effect and save the R&D time and cost, boosting investment in innovation [11]. However, if enterprises are overly agglomerated, productivity of companies will decrease while the widely shared knowledge and its spillover effect may lead to lower innovation income inside the company, therefore, enterprises will cut innovation investment [12]. Accordingly, the transmission of economic agglomeration confirms the inverted U-shaped relationship between local government debt and enterprise innovation.

Land transfer fee is one of local governments’ important sources to pay debts, and land prices partially determin housing prices. To sum up, local government debt may lead to higher housing prices [13]. Reasonable housing price rise will enhance the real estate mortgage effect and ease the financing constraint on enterprises, facilitating enterprise innovation [14]. However, skyrocketing housing price will, on the one hand, lead to great financing constraint on enterprises other than property developers, and, on the other hand, enterprise investments will flood into property developers as home market’s attractive profits outperform other manufacturers and service providers [15], thus inhibiting enterprise innovation. Accordingly, the transmission of a rising housing price leads to an inverted U-shaped relationship between local government debt and enterprise innovation.

To sum up, this paper makes Hypothesis 1: the relationship between local government debt and enterprise innovation follows an inverted U-shaped pattern.

## 3. Research design

### 3.1. Variable selection and description

1. Explained variable. Since innovation usually produce uncertain results, investment in innovation could serve as an effective indicator reflecting an enterprise’s willingness to innovate. Accordingly, the share (Rdoperate) of R&D investment accounting for operation revenue is used as a parameter to measure enterprises innovation. Smith [16] pointed out that R&D expens do not cover human resource development, technology introduction, digestion and absorption, representing only a small part of enterprise innovation, so he suggested using the ratioof increased intangible assets to total assets (Iasset) to reflect enterprises innovation. Additionally, the log of R&D expenditure plus 1 (InRd) is used to reflect enterprises innovation.
2. Core explanatory variable. Local government debt (Debtgdp) is measured by the ratio of a city’s debt balance from the Wind database to GDP.
3. Mediating variable. Enterprise financing constraint (SA), is measured by the good exogenous SA index, referring to Hadlock and Pierce [17]. Enterprise profit (Profit) is measured by the ratio of total enterprise profit to enterprise operation income.
4. Moderating variable. Digital finance (Dif) is measured by the *Digital Finance Inclusive Finance Index* compiled by the Research Center for Internet Finance, Peking University.
5. Control variable. To avoid variable omission, the model selects control variables at enterprise and city level, including return on assets (Roa, the ratio of net profit to total assets), leverage ratio (Leverage, the ratio of total debt to total assets), enterprise size (Insize, the log of total assets), operation income growth rate (Oig), government subsidy (Subsidy, the ratio of government subsidy to total assets), shareholding ratio (Top1, the shareholding ratio of the largest shareholder), independent director ratio (Inddir, the ratio of the total number of independent directors to the total number of directors), combined title of Board Chair and CEO (Dual), Tobin’s Q ratio (Tobinq), regional GDP (Inpgdp, the log of regional GDP per capita).

### 3.2. Model specification and empirical strategy

Eq (1) is constructed to test the inverted U-shaped relationship between local government debt and enterprise innovation, referring to Lind and Mehlum [18]:

$${Innova}_{c,i,t}=\beta_{0}+\beta_{1}{Debtgdp}_{c,t}+\beta_{2}{Debtgdp2}_{c,t}+\sum \varphi {Controls}_{c,i,t}+\sum \gamma Year+\sum \lambda Ind+\varepsilon_{c,i,t}$$ (1)

Where Innova is enterprise innovation, Debtgdp, Debtgdp2 are the core explanatory variable local government debt and its square term, Control represents control variable including return on assets, leverage ratio, enterprise size, operation income growth rate, government subsidy, shareholding ratio, independent director ratio, combined title of Board Chair and CEO, Tobin’s Q ratio, regional GDP,Year represents the time-fixing effect,Ind represents the industry-fixing effect, *ε* is random error terms. If the empirical results show that the coefficients of Debtgdp and Debtgdp2 are significantly positive and negative respectively, there will be an inverted U-shaped relationship between local government debt and enterprise innovation. The empirical analysis employs robust standard errors for all models by default, controlled time and industry fixed effects.

To clarify the internal mechanism behind the impact of local government debt on enterprise innovation, Eqs (2) and (3) are built using the mediating effect test method proposed by Baron [19]:

$${Mediator}_{c,i,t}=\beta_{0}+\beta_{1}{Debtgdp}_{c,t}+\beta_{2}{Debtgdp2}_{c,t}+\sum \varphi {Controls}_{c,i,t}+\sum \gamma Year+\sum \lambda Ind+\varepsilon_{c,i,t}$$ (2)

$${Innova}_{c,i,t}=\beta_{0}+\beta_{1}{Debtgdp}_{c,t}+\beta_{2}{Debtgdp2}_{c,t}+\beta_{3}{Mediator}_{c,i,t}+\sum \varphi {Controls}_{c,i,t}+\sum \gamma Year+\sum \lambda Ind+\varepsilon_{c,i,t}$$ (3)

Where the Mediator represents mediating variable, including financing constraint (SA) and enterprise profit (Profit), Innova is enterprise innovation, Debtgdp, Debtgdp2 are the local government debt and its square term, Control represents control variable and the specific variable it contain is described in detail in Eq (1). Year represents a time-fixing effect, Ind represents the industry-fixing effect, *ε* is random error terms.If the coefficient of core explanatory variable in Eq (1) is significant, regression can be performed in Eq (2). Eq (2) is used to test whether the nonlinear relationship between mediating variable and local government debt is significant, and if it is significant, the regression can be performed in Eq (3). In Eq (3), if the symbols of coefficients of Debtgdp and Debtgdp2 are the same and significant as in Eq (1), the coefficient of mediating variable is significant, and the model fitting improved, there is an mediating effect.

To further analyze the moderating effect of digital finance, Eq (4) is built, referring to Haans [20]:

$${Innova}_{c,i,t}=\beta_{0}+\beta_{1}{Debtgdp}_{c,t}+\beta_{2}{Debtgdp2}_{c,t}+\beta_{3}{Adjust}_{c,i,t}\times {Debtgdp}_{c,i,t}+\beta_{4}{Adjust}_{c,i,t}\times {Debtgdp2}_{c,i,t}+\beta_{5}{Adjust}_{c,i,t}+\sum \varphi {Controls}_{c,i,t}+\sum \gamma Year+\sum \lambda Ind+\varepsilon_{c,i,t}$$ (4)

Where Innova is enterprise innovation, Debtgdp, Debtgdp2 are the local government debt and its square term, Adjust is regulation Variables, which stands for digital finance in this article, Control represents control variable, which contains the same specific variables as Eq (1), Year represents the time-fixing effect, Ind represents the industry-fixing effect, *ε* is random error terms. If coefficients β_3_ and β_4_ in Eq (4) are significant, digital finance will play a moderating effect. Referring to Cohen et al. [21], if the symbols of coefficients of β_3_ and β_4_ are the same as those of β_1_ and β_2_, respectively, digital finance enhance the impact of local government debt on enterprise innovation; if the symbols are the opposite, digital finance will weaken the impact of local government debt on enterprise innovation.

### 3.3. Data source and description

"Digital Financial Inclusion Index" published since 2011,the land transfer data was updated to 2017, and the new "Budget Law" enacted in 2015, requiring the stripping of LGFV’s financing functions, but the transformation of the LGFV is slow. In June 2018, the Ministry of Finance launched a new round of work to resolve hidden debts, according to Wind database statistics, By the end of 2018, local governments had exchanged debt through bond issuance, accounting for 85 percent of the existing debt borrowed through LGFV and other means at the end of 2014. Therefore, in order to ensure the coherence and reliability of the data, 2011–2017 is the time range of this study. To make a robust conclusion, the model removes all financial listed companies, ST enterprises and data omitted listed companies out of the sample base, leaving a total of 11,539 observed values to be used. Enterprise data in this paper are extracted from CSMAR and Choice databases, debt data are from Wind database, and other data are from China City Statistical Yearbook. To eliminate the effect of outliers, the 1% and 99% quantiles of continuous variables are winsorized. The data characteristics of correlation variables are listed in Table 1. The means of three innovation indicators are 0.0871, 0.00545 and 16975, respectively, and the standard deviations are 4.447, 0.0206 and 83136, respectively, indicating significant differences in the innovation of enterprises. Similarly, the mean value of debt is 0.195, and the standard deviation is 0.286, indicating significant differences in the local government debt of enterprises. The statistical results of other control variables are highly consistent with existing relevant literature.

**Table 1:** Descriptive statistics.

Variable	Sample size	Mean value	Standard deviation	Minimum	25% quartile	Median	75% quartile	Maximum
Rdoperat	11539	0.0871	4.447	0	0.0191	0.0351	0.0527	477.7
Iasset	11539	0.00545	0.0206	-0.738	-0.00104	0.000245	0.00669	0.544
Rd	11539	16975	83136	0	2089	4668	10765	5618867
Debtgdp	11539	0.195	0.286	0.00121	0.0272	0.0722	0.292	2.921
Oig	11539	0.179	0.860	-0.872	-0.00802	0.117	0.266	71.23
Insize	11539	12.76	1.261	9.871	11.86	12.57	13.42	19.30
Leverage	11539	0.381	0.199	0.00752	0.219	0.363	0.527	0.979
Roa	11539	0.0456	0.0517	-0.683	0.0183	0.0428	0.0717	0.340
Top1	11539	35.07	14.70	2.197	23.62	33.53	44.81	89.09
Inddir	11539	0.375	0.0554	0.182	0.333	0.333	0.429	0.800
Tobinq	11539	2.990	2.213	0.219	1.603	2.324	3.622	34.01
Inpgdp	11539	11.38	0.521	9.219	11.06	11.48	11.73	13.06
Dual	11539	0.300	0.458	0	0	0	1	1
Subsidy	11539	0.00530	0.00728	0	0.00170	0.00349	0.00650	0.234
Landgdp	11539	0.0705	0.0632	0.00251	0.0370	0.0537	0.0895	0.719

## 4. Empirical results and economic interpretation

### 4.1. Benchmark regression results

Enterprise innovation items listed in columns 1 and 2 in Table 2 are measured by Rdoperate. Column 2 incorporates control variables. Accordingly, both the primary and quadratic term coefficients of local government debt are significant at 1% level, and the inflection point of local government debt is 0.689 (the inflection point of quadratic function is approximately equal to the negative number of the result of primary term coefficient divided by twice the quadratic term coefficient). The minimum and the maximum values of local government debt are 0.00121 and 2.921, respectively, suggesting the inverted U-shaped relationship between local government debt and enterprise innovation. Enterprise innovation items in columns 3 and 4 are measured by Iasset, and those in columns 5 and 6 are measured by InRd. Estimating the debt inflection points in columns 4 and 6 seperately, it is found that the inverted U-shaped pattern remains between local government debt and enterprise innovation, which verifies the Hypothesis 1. The control variables indicates that good profitability, large enterprises, small agent levels, large market value, developed regions and government subsidy promote enterprise innovation, while high debt ratio and ownership concentration are obstacles.

**Table 2:** Regression results of local government debt impacting enterprise innovation.

Variable	Rdoperat		Iasset		InRd	
	(1)	(2)	(3)	(4)	(5)	(6)
Debtgdp	0.0478***	0.0393***	0.00593***	0.00549***	1.253***	0.254**
	[0.0048]	[0.0044]	[0.0015]	[0.0015]	[0.1447]	[0.1059]
Debtgdp2	-0.0368***	-0.0285***	-0.00412***	-0.00361**	-0.998***	-0.289***
	[0.0044]	[0.0040]	[0.0014]	[0.0014]	[0.1400]	[0.1045]
Oig		-0.00158		0.00788***		0.0815**
		[0.0013]		[0.0006]		[0.0344]
Insize		0.00186***		0.000587***		0.814***
		[0.0004]		[0.0002]		[0.0128]
Leverage		-0.0577***		-0.00553***		-0.231***
		[0.0026]		[0.0010]		[0.0714]
Roa		-0.127***		-0.00000788		2.824***
		[0.0108]		[0.0035]		[0.2546]
Top1		-0.000264***		-0.0000202**		-0.00231***
		[0.0000]		[0.0000]		[0.0007]
Dual		0.00388***		0.000860***		0.0383**
		[0.0008]		[0.0003]		[0.0189]
Inddir		0.0235***		0.00501*		-0.114
		[0.0061]		[0.0026]		[0.1728]
Tobinq		0.00395***		0.0000181		0.0150**
		[0.0003]		[0.0001]		[0.0061]
Inpgdp		0.00891***		-0.000352		0.315***
		[0.0006]		[0.0003]		[0.0202]
Subsidy		1.058***		0.126***		1.927***
		[0.0932]		[0.0329]		[0.208]
Cons	0.00859***	-0.0966***	0.00978***	0.00473	6.615***	-7.407***
	[0.0021]	[0.0086]	[0.0016]	[0.0041]	[0.1289]	[0.3194]
Time fixed effect	YES	YES	YES	YES	YES	YES
Industry fixed effect	YES	YES	YES	YES	YES	YES
Sample size	11539	11539	11539	11539	11539	11539
R-square	0.2225	0.3563	0.215	0.523	0.1036	0.5058

Note: (1) The values in parentheses are robust standard errors; (2) Mean centering of the secondary term is performed; (3) * * *, * * * and * indicated that the values are significant at 1%, 5% and 10% levels, respectively. The same as below, so we will no longer repeat.

### 4.2. Endogeneity processing

There may be a causal relationship between local government debt and enterprise innovation. Local government debt impacts enterprise innovation, in other words, local government may raise loans to subsidize enterprises for innovation. Instrumental variables (IVs), including official promotion incentive (Promotion) and terrain relief (Terrain), are used to solve the possible endogeneity issues of the model. Endogenous explanatory variables included the primary and secondary terms of local government debt.

Promotion meets the correlation and objectivity requirements of IVs. As the subjective behavior of government officials, Promotion boosts local government debt expansion [22, 23], but it cannot affect the innovative decisions made by enterprises. Promotion is calculated based on the method proposed by Cheng [24].

Terrain decides the supply of land, thus impacting land transfer income. Land transfer income serves as a major source of debt repayment fund for local governments, and it also determines the size of local government debt as the basis of mortgage loan amount limit. Terrain is closely correlated to local government debt. In terms of objectivity, terrain is naturally formed, which has no influence on an enterprise’s innovation decisions-making. As a result, terrain meets the requirements of IVs, so we calculated the Terrain of each city using the method proposed by Lin and Tan [25].

The model is estimated via 2SLS, and the regression results are shown in Table 3. The F of regression results at stage 1 are 73.44 and 70.27, respectively, indicating a strong correlation between IVs and endogenous explanatory variables. The Cragg-Donald Wald F statistic in regression results at stage 2 is 27.19, greater than the critical value of 4.58, indicating that there is no weak IV problem. The primary term coefficient of local government debt is significantly positive, and the quadratic term coefficient is significantly negative, suggesting that there is an inverted U-shaped relationship between local government debt and enterprise innovation, verifying the Hypothesis 1.

**Table 3:** Endogeneity test: Regression results via 2SLS.

Variable	Rdoperat		
	Stage 1	Stage 1	Stage 2
Debtgdp			0.175***
			[0.0415]
Debtgdp2			-0.187***
			[0.0474]
Promotion	0.000436	0.00959***	
	[0.0027]	[0.0029]	
Terrain	0.130***	0.114***	
	[0.0067]	[0.0077]	
Oig	-0.00356	-0.00275	-0.00154
	[0.0083]	[0.0089]	[0.0014]
Insize	0.0141***	0.00567**	0.000856
	[0.0026]	[0.0028]	[0.0005]
Leverage	-0.0194	0.0126	-0.0532***
	[0.0143]	[0.0150]	[0.0031]
Roa	-0.00995	-0.0174	-0.129***
	[0.0553]	[0.0590]	[0.0115]
Top1	0.000239	0.0000673	-0.000286***
	[0.0002]	[0.0002]	[0.0000]
Dual	-0.0179***	-0.0172***	0.00359***
	[0.0045]	[0.0047]	[0.0008]
Inddir	0.0262	0.0366	0.0254***
	[0.0418]	[0.0464]	[0.0068]
Tobinq	0.00317**	0.00104	0.00369***
	[0.0015]	[0.0015]	[0.0003]
Inpgdp	0.0791***	0.0542***	0.00580***
	[0.0039]	[0.0038]	[0.0012]
Subsidy	2.047***	1.638***	1.033***
	[0.4094]	[0.4531]	[0.0976]
Cons	-1.069***	-0.835***	-0.0707***
	[0.0599]	[0.0622]	[0.0127]
Time fixed effect	YES	YES	YES
Industry fixed effect	YES	YES	YES
N	11539	11539	11539
F	73.44	70.27	107.47
R-square	0.2818	0.1929	0.227
Cragg-Donald Wald F statistic	——	——	27.19

### 4.3. Robustness test

To ensure the conclusion to be reliable, this paper conduct the robustness test from the following four aspects:

1. Use the previous explained variable. Considering the possible simultaneous bias between local government debt and enterprise innovation, regression of the previous explained variable is performed. The first 3 columns of Table 4 show that the relationship between local government debt and enterprise innovation is still following an apparent inverted U-shaped pattern after using the previous explained variable.
2. Replace the explained variable. The ratio (Rdasset) of R&D investment to total assets was used to express enterprise innovation. The column 4 of Table 4 confirms that the relationship between local government debt and enterprise innovation can still be denoted with an inverted U-shaped curve after replacing the explained variable.
3. Change the regression model. The minimum value of Rdoperat is 0, which is a left truncated datum, so Tobit model performs the regression. The regression results in column 5 of Table 4 shows that the conclusions remains verified after changing the regression model.
4. Replace the core explanatory variable. Land transfer income serves as one of major fund resources for debt repayment made by local governments, closely correlating to debt size [26–28]. The ratio (Landgdp) of land transfer income to GDP replaces the local government debt for regression. The regression results in column 6 of Table 4 show that conclusions remain unchanged after replacing the core explanatory variable.

**Table 4:** Regression results of robustness test.

Variable	(1)	(2)	(3)	(4)	(5)	(6)
	Rdoperat_t+1_	Isaaet_t+1_	InRd_t+1_	Rdasset	Rdoperat	Rdoperat
Debtgdp	0.0522***	0.00978***	0.323**	0.00710***	0.0393***	
	[0.0063]	[0.0021]	[0.1508]	[0.0017]	[0.0036]	
Debtgdp2	-0.0438***	-0.00885***	-0.359*	-0.00631***	-0.0285***	
	[0.0074]	[0.0024]	[0.2010]	[0.0016]	[0.0035]	
Landgdp						0.0988***
						[0.0196]
Landgdp2						-0.315***
						[0.0726]
Oig	-0.000883	0.00225***	0.318***	-0.000449	-0.00158	-0.00158
	[0.0015]	[0.0006]	[0.0394]	[0.0005]	[0.0012]	[0.0013]
Insize	0.00156***	-0.000119	0.801***	-0.000843***	0.00186***	0.00227***
	[0.0005]	[0.0002]	[0.0153]	[0.0002]	[0.0004]	[0.0004]
Leverage	-0.0564***	-0.00411***	-0.149*	0.00220**	-0.0577***	-0.0587***
	[0.0030]	[0.0011]	[0.0813]	[0.0010]	[0.0023]	[0.0026]
Roa	-0.0771***	0.0246***	3.133***	0.0451***	-0.127***	-0.130***
	[0.0116]	[0.0039]	[0.3009]	[0.0042]	[0.0085]	[0.0109]
Top1	0.000286***	0.0000309***	-0.00273***	0.0000521***	0.000264***	0.000256***
	[0.0000]	[0.0000]	[0.0008]	[0.0000]	[0.0000]	[0.0000]
Dual	0.00362***	0.00123***	0.0342	0.000327	0.00388***	0.00361***
	[0.0009]	[0.0004]	[0.0217]	[0.0003]	[0.0007]	[0.0008]
Inddir	0.0263***	0.00573*	-0.0541	0.000849	0.0235***	0.0243***
	[0.0071]	[0.0030]	[0.1964]	[0.0026]	[0.0061]	[0.0062]
Tobinq	0.00361***	0.000169	0.0254***	0.00118***	0.00395***	0.00405***
	[0.0004]	[0.0001]	[0.0071]	[0.0001]	[0.0002]	[0.0003]
Inpgdp	0.00773***	-0.000138	0.292***	0.00483***	0.00891***	0.00987***
	[0.0007]	[0.0003]	[0.0224]	[0.0003]	[0.0007]	[0.0006]
Subsidy	1.061***	0.0382	1.728***	0.469***	1.058***	1.086***
	[0.1109]	[0.0332]	[0.237]	[0.0380]	[0.0635]	[0.0936]
Cons	-0.0808***	0.0110**	-6.786***	-0.0426***	-0.0966***	-0.115***
	[0.0100]	[0.0047]	[0.3718]	[0.0040]	[0.0096]	[0.0086]
Time fixed effect	YES	YES	YES	YES	YES	YES
Industry fixed effect	YES	YES	YES	YES	YES	YES
N	8495	8495	8495	11539	11539	11539
R-square	0.3585	0.514	0.5018	0.2851	——	0.3502
Log Likelihood	——	——	——	——	22520.457	——

### 4.4. Heterogeneity analysis

The above sections analyze the general impact of local government debt on enterprise innovation, but ignored the heterogeneity of such impact. To solve this issue, enterprises are cataogrized into large and small enterprises based on enterprise scale (total enterprise assets), SOEs and non-SOEs according to enterprise attributes, while regions are grouped as high financial level and low financial level based on local financial development (the ratio of regional bank loans to GDP), so as to test the differences between regions for the impact of local government debt on enterprise innovation. The regression results are shown in Table 5.

**Table 5:** Regression results of heterogeneity analysis.

Rdoperat						
Variable	Small enterprises	Large enterprises	Non-SOEs	SOEs	Regions with low financial level	Regions with high financial level
Debtgdp	0.0448***	0.0158***	0.00997*	0.0556***	0.0225***	0.0436***
	[0.0051]	[0.0059]	[0.0059]	[0.0058]	[0.0070]	[0.0060]
Debtgdp2	-0.0318***	-0.0155***	-0.0123**	-0.0375***	-0.0175***	-0.0303***
	[0.0047]	[0.0051]	[0.0050]	[0.0055]	[0.0062]	[0.0052]
Oig	-0.00216	-0.000953	0.0000438	-0.00265*	-0.00333**	-0.000414
	[0.0014]	[0.0021]	[0.0025]	[0.0015]	[0.0015]	[0.0021]
Insize	0.00390***	-0.000577	-0.000673	0.00430***	0.00111**	0.00247***
	[0.0007]	[0.0007]	[0.0006]	[0.0006]	[0.0005]	[0.0006]
Leverage	-0.0641***	-0.0175***	-0.0216***	-0.0742***	-0.0452***	-0.0716***
	[0.0029]	[0.0051]	[0.0034]	[0.0034]	[0.0032]	[0.0042]
Roa	-0.138***	-0.0487***	-0.0263*	-0.176***	-0.0946***	-0.167***
	[0.0121]	[0.0188]	[0.0156]	[0.0139]	[0.0129]	[0.0178]
Top1	-0.000294***	-0.0000824**	-0.000109***	-0.000251***	-0.000166***	-0.000368***
	[0.0000]	[0.0000]	[0.0000]	[0.0000]	[0.0000]	[0.0000]
Dual	0.00434***	-0.000934	-0.0016	0.00379***	0.00288***	0.00478***
	[0.0008]	[0.0015]	[0.0013]	[0.0009]	[0.0009]	[0.0013]
Inddir	0.0280***	0.00661	0.00912	0.0280***	0.0145*	0.0321***
	[0.0073]	[0.0084]	[0.0083]	[0.0079]	[0.0075]	[0.0099]
Tobinq	0.00419***	0.00442***	0.00340***	0.00426***	0.00320***	0.00448***
	[0.0003]	[0.0012]	[0.0008]	[0.0004]	[0.0004]	[0.0005]
Inpgdp	0.0103***	0.000782	0.00515***	0.0108***	0.00739***	0.0131***
	[0.0007]	[0.0011]	[0.0009]	[0.0008]	[0.0007]	[0.0015]
Subsidy	1.138***	0.609***	0.610***	1.374***	0.940***	1.184***
	[0.1032]	[0.1506]	[0.1470]	[0.1152]	[0.1070]	[0.1582]
Cons	-0.135***	0.013	-0.0278**	-0.151***	-0.0736***	-0.143***
	[0.0116]	[0.0145]	[0.0120]	[0.0116]	[0.0104]	[0.0183]
Time fixed effect	YES	YES	YES	YES	YES	YES
Industry fixed effect	YES	YES	YES	YES	YES	YES
N	9823	1716	3562	7977	6316	5223
R-square	0.3392	0.2818	0.2600	0.3718	0.3046	0.3983

The estimation of the inflection point of local government debt for large and small enterprises show that large enterprises have a lower inflection point. Compared with small enterprises, large enterprises can get obtain bank loans more easily, which is the main source of local government debt. An increasing local government debt amount will crowd out the bank credit resources and improve the level of financing constraint for large enterprises. SOEs have a higher inflection point of local government debt than that of non-SOEs, because the former type of companies are usually the first choice of financial institutions when issuing loans due to the government guarantee backing SOEs [29–31]. Some SOEs can finance directly, so the financing constraint on these enterprises are relative loose. In comparison with low financial regions, the inflection point of local government debt for regions with high financial level is higher because higher financial level means more funds available for enterprises and less financing constraint on enterprises.

## 5. Mechanistic identification and test

The above research conducts an empirical test on the impact of local government debt on enterprise innovation and analyzes the heterogeneity of such impact. However, the transmission path has not been clarified. This chapter will analyze the impact mechanism from the two aspects of enterprise profit and financing constraint.

### 5.1. Analysis of the mediating effect based on enterprise profit

Local government debt will promote economic growth and moderate economic agglomeration if the debt scale is at an appropriate level, which in turn increases enterprise profit. Moderate housing price rise drives the price of other related products. On the one hand, this enhances the real estate’s mortgage effect, reduces the financing cost of enterprises, and increases enterprise profit. On the other hand, high local government debt will hinder economic growth overwhelmed by excessive economic agglomeration, thus decreasing enterprise profits. The surging housing price will increase enterprise operating expenditure and undermine enterprise profitability. The R&D cycle of innovation activities is time-consuming, uncertain and has a large demand for capital investment. The innovation cost is usually paid by the internal capital of enterprise for strategic reasons [32]. Meahwhile, an enterprise’s internal capital denpends on operation profit. Therefore, it can be concluded that local government debt’s impact on enterprise innovation is mediated by business profit. Table 6 illustrates the regression results of enterprise profit as the mediating variable.

**Table 6:** Regression results of enterprise profit as the mediating variable.

Rdoperat				
Variable	(1)	(2)	(3)	(4)
	Rdoperat	Profit	Rdoperat	Rdoperat_t+1_
Debtgdp		0.0348***	0.0363***	0.0472***
		[0.0078]	[0.0043]	[0.0060]
Debtgdp2		-0.0202***	-0.0268***	-0.0404***
		[0.0074]	[0.0040]	[0.0070]
Profit	0.0881***		0.0853***	0.116***
	[0.0068]		[0.0067]	[0.0078]
Oig	-0.00228*	0.00737***	-0.00221*	-0.0019
	[0.0013]	[0.0027]	[0.0013]	[0.0015]
Insize	0.00149***	0.00891***	0.00110***	0.000554
	[0.0004]	[0.0008]	[0.0004]	[0.0005]
Leverage	-0.0469***	-0.134***	-0.0463***	-0.0405***
	[0.0027]	[0.0050]	[0.0027]	[0.0030]
Roa	-0.291***	1.853***	-0.285***	-0.290***
	[0.0147]	[0.0234]	[0.0146]	[0.0175]
Top1	-0.000236***	-0.000228***	-0.000245***	-0.000265***
	[0.0000]	[0.0000]	[0.0000]	[0.0000]
Dual	0.00294***	0.00771***	0.00323***	0.00277***
	[0.0008]	[0.0015]	[0.0008]	[0.0009]
Inddir	0.0199***	0.0409***	0.0200***	0.0212***
	[0.0061]	[0.0119]	[0.0061]	[0.0069]
Tobinq	0.00392***	0.00171***	0.00381***	0.00342***
	[0.0003]	[0.0005]	[0.0003]	[0.0004]
Inpgdp	0.0102***	-0.00349***	0.00921***	0.00807***
	[0.0006]	[0.0013]	[0.0006]	[0.0007]
Subsidy	1.103***	-0.151	1.071***	1.065***
	[0.0917]	[0.1401]	[0.0915]	[0.1073]
Cons	-0.111***	0.0245	-0.0987***	-0.0818***
	[0.0084]	[0.0192]	[0.0085]	[0.0099]
Time fixed effect	YES	YES	YES	YES
Industry fixed effect	YES	YES	YES	YES
N	11539	11539	11539	8495
R-square	0.3681	0.6931	0.3744	0.3905

The column 1 of Table 6 shows that improving enterprise profit can significantly promote enterprise innovation. The significant inverted U-shaped relationship between local government debt and current enterprise innovation and previous enterprise innovation has been shown in column 2 of Table 2 and column 1 of Table 4, so regression of Eq (2) can be performed. As the regression results of Eq (2) in column 2 of Table 6 show, as local government debt expands, enterprise profit first increases before entering a dropping phase, indicating the inverted U-shaped relationship between local government debt and enterprise profit, so regression of Eq (3) can be performed. The regression results of Eq (3) are shown in columns 3 and 4 of Table 6, which are the regression results of current enterprise innovation and previous enterprise innovation, respectively. Compared with column 2 of Table 2, in column 3 of Table 6, the significance of the model has been improved (adjusted R-square), while the symbols of primary and secondary term coefficients of local government debt shows a slight change. The profit coefficient is also significant, indicating that enterprise profit plays a mediating effect in local government debt impacting enterprise innovation. In comparison with column 1 of Table 4, the regression results of previous enterprise innovation in column 4 of Table 6 show that the partial mediating effect of enterprise profit remains after considering the possible simultaneous bias of the model. Local government debt impacted enterprise innovation through enterprise profit, thus forming a transmission path of local government debt—enterprise profit—enterprise innovation.

### 5.2. Analysis of the mediating effect based on financing constraint

Moderate debt scale can boost enterprise profit, increase the liquidity and ease financing constraints, thus driving enterprise innovation, while excessive local government debt will hinder the growth of profitability and tighten the internal financing constraint on enterprises. To make things worse, overly high local government debt will crowd out the bank credit resources and make debt financing constraints undertaken by large enterprises severer, thus impeding enterprise innovation. Thus, local government debt will impact enterprise innovation through financing constraint. Table 7 demonstrates the regression results of financing constraint as the mediating variable.

**Table 7:** Regression results of financing constraint as the mediating variable.

Rdoperat				
Variable	(1)	(2)	(3)	(4)
	Rdoperat	SA	Rdoperat	Rdoperat_t+1_
Debtgdp		0.152***	0.0373***	0.0498***
		[0.0200]	[0.0044]	[0.0063]
Debtgdp2		-0.109***	-0.0271***	-0.0419***
		[0.0189]	[0.0040]	[0.0074]
SA	0.0145***		0.0131***	0.0121***
	[0.0015]		[0.0015]	[0.0018]
Oig	-0.00242*	0.0548***	-0.00230*	-0.00164
	[0.0013]	[0.0067]	[0.0013]	[0.0015]
Insize	0.00393***	-0.114***	0.00334***	0.00288***
	[0.0004]	[0.0023]	[0.0004]	[0.0005]
Leverage	-0.0570***	-0.117***	-0.0562***	-0.0546***
	[0.0026]	[0.0137]	[0.0026]	[0.0030]
Roa	-0.125***	-0.217***	-0.124***	-0.0733***
	[0.0109]	[0.0491]	[0.0108]	[0.0116]
Top1	-0.000284***	0.00195***	-0.000290***	-0.000310***
	[0.0000]	[0.0001]	[0.0000]	[0.0000]
Dual	0.00315***	0.0312***	0.00348***	0.00330***
	[0.0008]	[0.0040]	[0.0008]	[0.0009]
Inddir	0.0206***	0.206***	0.0208***	0.0240***
	[0.0062]	[0.0352]	[0.0061]	[0.0071]
Tobinq	0.00398***	0.00679***	0.00386***	0.00352***
	[0.0003]	[0.0014]	[0.0003]	[0.0004]
Inpgdp	0.0101***	-0.0125***	0.00907***	0.00794***
	[0.0006]	[0.0038]	[0.0006]	[0.0007]
Subsidy	1.078***	0.849**	1.047***	1.057***
	[0.0933]	[0.3546]	[0.0930]	[0.1107]
Cons	-0.0824***	-1.869***	-0.0722***	-0.0584***
	[0.0086]	[0.0551]	[0.0087]	[0.0102]
Time fixed effect	YES	YES	YES	YES
Industry fixed effect	YES	YES	YES	YES
N	11539	11539	11539	8495
R-square	0.3529	0.4793	0.3597	0.3612

The column 1 of Table 7 shows an apparent positive relationship between financing constraint and enterprise innovation. According to the table, the lower level of financing constraint faced by enterprises is more helpful for enterprise innovation. The significant inverted U-shaped relationship between local government debt and current enterprise innovation and previous enterprise innovation has been given in column 2 of Table 2 and column 1 of Table 4, so regression of Eq (2) can be performed. As the regression results of Eq (2) shown in column 2 of Table 7, when local government debt scale increases, the financing constraint on enterprises drops before bouncing back to rise, indicating a significant inverted U-shaped relationship between local government debt and enterprise profit, so the regression of Eq (3) can be performed. Columns 3 and 4 in Table 7 show the regression results of Eq (3) of current enterprise innovation and previous enterprise innovation, respectively. Compared with column 2 of Table 2, the significance of the model is improved (adjusted R-square) in column 3 of Table 7, the symbols of primary and secondary term coefficients of local government debt only change slightly. The SA coefficient is also significant, indicating that the impact local government debt on enterprise innovation can be partially mediated by financing constraints. The column 4 of Table 7 shows the regression results of the previous enterprise innovation. In comparison with column 1 of Table 4, the partial mediating effect of financing constraint in column 4 of Table 7 remains considering the possible simultaneous bias of the model. Local government debt impacts enterprise innovation through financing constraint, forming a transmission path of local government debt—enterprise financing constraint—enterprise innovation.

## 6. Further analysis

Digital finance, an innovative financial model, expands the service scope of traditional finance. At the same time, the convenient and efficient approval process reduces the financing cost of enterprises [33]. Would the advantage of digital finance be reflected in the impact of local government debt on enterprise innovation? To explore in to this aspect, this paper conducts an empirical test on the moderating effect of digital finance.

The column 1 of Table 8 shows that digital finance promotes enterprise innovation because it expands the service scope of finance, eases financing constraint on enterprises, thus driving enterprise innovation. In column 2, which adds local government debt and its square based on column 1, there is still a significant inverted U-shaped relationship between local government debt and enterprise innovation, with the symbol of digital finance as significant as that in column 1. In column 3 where no control variable added, there is a significant inverted U-shaped relationship between local government debt and enterprise innovation, in which β_3_ is significantly negative while β_4_ and β_5_ are significantly positive, indicating that digital finance plays a mediating effect in the impact of local government debt on enterprise innovation. In column 4, where control variable is added based on column 3, it shows that the inverted U-shaped relationship between local government debt and enterprise innovation exists with the symbols of β_3_, β_4_ and β_5_ the same and significant as in column 3. The impact of digital finance on the curve form can be simply determined by the symbol of β_4_. As the β_4_ is greater than 0, the higher the level of digital finance, the flatter the curve between local government debt and enterprise innovation. The impact of digital finance on the inflection point of the curve is determined by the value of β_1_β_4_−β_2_β_3_. Since the value is greater than 0, a higher level of digital finance leads the inflection point of the impact of local government debt on enterprise innovation moving further right. The impact of digital finance on the overall level of the curve is determined by the value of β_4_ and β_3_^2^−4β_4_β_5_. The β_4_ is greater than 0, and β_3_^2^−4β_4_β_5_ less than 0, so digital finance improves the overall level of the curve between local government debt and enterprise innovation. To sum up, the high level digital finance makes the inverted U-shaped relationship between local government debt and enterprise innovation flatter, in other words reducing the enterprises innovation volatility caused by local government debt. An advanced digital finance industry also makes the inflection point of local government debt impacting enterprise innovation move right, or enables enterprises to adapt to higher debt level. In conclusion, digital finance plays a mediating effect in the impact of local government debt on enterprise innovation.

**Table 8:** Regression results of digital finance as the mediating variable.

Rdoperat				
Vairiable	(1)	(2)	(3)	(4)
Debtgdp		0.0319***	0.0391***	0.0405***
		[0.0046]	[0.0066]	[0.0061]
Debtgdp2		-0.0231***	-0.0393***	-0.0356***
		[0.0041]	[0.0088]	[0.0080]
Dif*Debtgdp			-0.00394***	-0.00290**
			[0.0013]	[0.0012]
Dif*Debtgdp2			0.00466***	0.00329**
			[0.0016]	[0.0015]
Dif	0.00263***	0.00161***	0.00310***	0.00162***
	[0.0003]	[0.0003]	[0.0002]	[0.0003]
Oig	-0.00167	-0.00161		-0.00173
	[0.0013]	[0.0013]		[0.0013]
Insize	0.00219***	0.00187***		0.00184***
	[0.0004]	[0.0004]		[0.0004]
Leverage	-0.0584***	-0.0577***		-0.0575***
	[0.0026]	[0.0026]		[0.0026]
Roa	-0.131***	-0.129***		-0.129***
	[0.0109]	[0.0108]		[0.0108]
Top1	-0.000263***	-0.000268***		-0.000268***
	[0.0000]	[0.0000]		[0.0000]
Dual	0.00329***	0.00365***		0.00362***
	[0.0008]	[0.0008]		[0.0008]
Inddir	0.0229***	0.0231***		0.0232***
	[0.0061]	[0.0061]		[0.0061]
Tobinq	0.00399***	0.00392***		0.00391***
	[0.0003]	[0.0003]		[0.0003]
Inpgdp	0.00254***	0.00456***		0.00421***
	[0.0010]	[0.0010]		[0.0010]
Subsidy	1.079***	1.056***		1.058***
	[0.0930]	[0.0929]		[0.0930]
Constant	-0.0455***	-0.0596***	-0.0147***	-0.0556***
	[0.0107]	[0.0107]	[0.0031]	[0.0111]
Time fixed effect	YES	YES	YES	YES
Industry fixed effect	YES	YES	YES	YES
N	11539	11539	11539	11539
R-square	0.3536	0.3579	0.2390	0.3584

Note: (1) Mean centering of the interaction term is performed; (2) To avoid the impact of excessive digital finance value on the conclusions, this paper reduces the digital financial index by 10 times.

## 7. Conclusions and suggestions for policy-making

Based on the data of city’s debt balance and listed companies from 2011 to 2017,this paper conducts an empirical test on the impact of local government debt on enterprise innovation. The test finds that the relationship between local government debt and enterprise innovation follows an inverted U-shaped pattern. Endogeneity processing and robustness test are conducted, confirming the above conclusions. The inflection point of local government debt of large enterprises, non-SOEs and regions with low financial development level is lower than that of small enterprises, SOEs and regions with high financial development level. To be specific, local government debt impacted enterprise innovation through financing constraint and enterprise profit. Meanwhile, digital finance plays a mediating effect in local government debt impacting enterprise innovation.

According to the above conclusions, this paper proposes the following recommendations for policy-making. First, a moderate amount of local government debt can promote enterprise innovation, but when the scale of debt exceeds a reasonable threshold, it will inhibit enterprise innovation. Policymakers should improve the financing system of LGD, incorporate debt risks into the assessment indicators of officials, prevent local governments from blindly borrowing and excessively interfering in credit resources, so as to promote enterprise innovation and long-term sustainable economic development. Second, we should deepen the reform of the traditional financial system, gradually change the traditional financial system dominated by the banking industry, reform and develop the capital market, increase the proportion of direct financing of enterprises, meet the financing needs of enterprises and serve enterprise innovation. Third, digital finance as a new financial model that relies on the technologies of big data, artificial intelligence and cloud computing, with the characteristics of convenience, low cost and low threshold, can service small and medium-sized enterprise and innovation, so it is necessary to further promote the development of digital finance, broaden the field of digital financial services and the scope of services, At the same time, the supervision and guidance of digital finance should be strengthened to fully release the efficiency of digital finance.

Further research can be carried out from the following three aspects: First, for the study of LGD, the calculation of the size of the debt is a difficult point. The debt data used in the existing literature is mainly municipal bonds issued by LGFV or LGFV’s debt balance, but these data are only part of the LGD, not the whole debt, different ways can be considered to measure the size of debt. Second, this paper analyzes the impact of debt on innovation from the perspective of listed companies, and researchers can further analyze the impact of local government debt on innovation from the perspective of provinces or cities, which is helpful to understanding the regional differences in the impact of debt on innovation, and is advantageous for policymakers in different regions to combine regional realities and reasonably borrow debt. Third, the research object of this paper is listed companies, but the financing situation of listed companies is better than that of the majority of small and micro enterprises. Therefore, the research object can be further transferred to small and micro enterprises to analyze the impact of LGD on the innovation of small and micro enterprises.

## 8. Limitations and future study direction

First, for the study of LGD, the calculation of the size of the debt is a difficult point. The debt data used in the existing literature is mainly municipal bonds issued by LGFV or LGFV’s debt balance, but these data are only part of the LGD, not the whole debt, different ways can be considered to measure the size of debt. Second, this paper analyzes the impact of debt on innovation from the perspective of listed companies, and researchers can further analyze the impact of local government debt on innovation from the perspective of provinces or cities, which is helpful to understanding the regional differences in the impact of debt on innovation, and is advantageous for policymakers in different regions to combine regional realities and reasonably borrow debt. Third, the research object of this paper is listed companies, but the financing situation of listed companies is better than that of the majority of small and micro enterprises. Therefore, the research object can be further transferred to small and micro enterprises to analyze the impact of LGD on the innovation of small and micro enterprises.
